# Supplementary material for: Bacteria break through one-micrometer-square passages by flagellar wrapping
Source: Nat Commun. 2026 Jan 20;17:713. doi: 10.1038/s41467-025-67507-9 (PMC12820085; doi:10.1038/s41467-025-67507-9)
Supplement: Supplementary file 1 — Supplementary Information [file 41467_2025_67507_MOESM1_ESM.pdf]

Supplementary Information

for Bacteria break through one-micrometer-square passages by flagellar wrapping

Aoba Yoshioka[1], Yoshiki Y. Shimada[2], Toshihiro Omori[3], Naoki A. Uemura[1], Kazutaka Takeshita[4], Kota Ishigami[5], Hiroyuki Morimura[5], Maiko Furubayashi[5], Tetsuo Kan[2]*, Hirofumi Wada[6]*, Yoshitomo Kikuchi[5]*, Daisuke Nakane[1]*

[1]Department of Engineering Science, Graduate School of Informatics and Engineering, The University of Electro-Communications, Tokyo, Japan. [2]Department of Mechanical Engineering and Intelligent Systems, Graduate School of Informatics and Engineering, The University of Electro-Communications, Tokyo, Japan. [3]Department of Finemechanics, Tohoku University, Sendai, Japan. [4]Department of Biotechnology, Faculty of Bioresource Sciences, Akita Prefectural University, Akita, Japan. [5]Biomanufacturing Process Research Center, National Institute of Advanced Industrial Science and Technology (AIST), Sapporo, Japan. [6]Department of Physical Sciences, Ritsumeikan University, Shiga, Japan.

*Corresponding author: tetsuokan@uec.ac.jp, hwada@fc.ritsumei.ac.jp, y-kikuchi@aist.go.jp, dice-k@uec.ac.jp

This PDF file includes:

Supplementary Notes
Figs. S1 to S9
Tables S1 to S5
Supplementary References

Supplementary Notes

Supplementary Note 1: Numerical simulations of a bacterium swimming in a narrow tube

A. Problem setting and governing equations

Consider a bacterium swimming in a narrow tube of radius R. Assume that the tube is filled with an incompressible Newtonian fluid of density ρ and viscosity μ and that the tube length L is sufficiently long compared to the tube radius ($L >> R$). Due to the small size of bacteria, the typical particle Reynolds number can be less than 1[1] and the inertia of the flow can be neglected. Fluid motion is then governed by the viscous dominated Stokes equation, and the flow field at an any point $\boldsymbol{x}$ is given by the following boundary integral equation[2]:

$$\mathbf{v}(\mathbf{x}) = -\frac{1}{8\pi\mu}\int_{cell}\mathbf{J}(\mathbf{x},\mathbf{y})\cdot\mathbf{q}(\mathbf{y})dS(\mathbf{y}) - \frac{1}{8\pi\mu}\int_{wall}\mathbf{J}(\mathbf{x},\mathbf{y})\cdot\mathbf{q}^{w}(\mathbf{y})dS(\mathbf{y}), \tag{1}$$

where $\mathbf{q}$ and $\mathbf{q}^w$ are the viscous traction on the cell surface and tube wall, respectively. The first term on the right represents the flow produced by the bacterium and the second term represents the boundary conditions of the tube. $\mathbf{J}$ is the Green's function of Stokeslet, which is given by

$$\mathbf{J} = \frac{\mathbf{I}}{r} + \frac{\mathbf{r}\otimes\mathbf{r}}{r^3}, \tag{2}$$

where $\mathbf{r} = \mathbf{x} - \mathbf{y}$, $r = |\mathbf{r}|$, and $\mathbf{I}$ is the identity matrix. The bacterium is thought to swim either in the unwrapped mode, in which the flagellum helixes behind the bacterium, or in the wrapped mode, in which the flagellum wraps around the bacterium (Fig. S7b). From the state of zero inertia, it is assumed that the bacterium swims with no force and torque:

$$\int_{cell}\mathbf{q}\,dS(\mathbf{y}) = 0, \text{and } \int_{cell}\mathbf{q}\times\hat{\mathbf{r}}\,dS(\mathbf{y}) = 0\,, \tag{3}$$

where $\hat{\mathbf{r}} = \mathbf{y} - \mathbf{y}_g$ is the relative position vector of the cell surface from the center of gravity $\mathbf{y}_g$. It is also assumed that the flagellum is driven by the motor torque $\mathbf{M}_{\mathrm{m}}$ at the base of the flagellum and that the torque $\mathbf{M}_{\mathrm{m}}$ and the fluid resistance are balanced for bacterial swimming:

$$\mathbf{M}_{\mathrm{m}} + \int_{flagellum}\mathbf{q}\times\tilde{\mathbf{r}}\,dS(\mathbf{y}) = 0, \tag{4}$$

where $\tilde{\mathbf{r}} = \mathbf{y} - \mathbf{y}_b$ is the relative position from the base of the flagellum $\mathbf{y}_b$.

B. Swimmer model

The bacterial body is modelled as a cylinder of length H with spherical ends of diameter D (see Fig. S7a). The ratio of H to D is set to $H/D = 3$ in the flow simulation. Assuming that the bacterium is moving rigidly, consider the translational swimming velocity $\mathbf{U}$, the rotational angular velocity of the cell body $\mathbf{\Omega}$ and the angular velocity of the flagellum $\boldsymbol{\omega}$:

$$\begin{cases} \mathbf{v}(\mathbf{x}) = \mathbf{U} + \mathbf{\Omega} \times \hat{\mathbf{r}} & \text{when } \mathbf{x} \in \text{cell body} \\ \mathbf{v}(\mathbf{x}) = \mathbf{U} + \mathbf{\Omega} \times \hat{\mathbf{r}} + \boldsymbol{\omega} \times \tilde{\mathbf{r}} & \text{when } \mathbf{x} \in \text{flagellum} \end{cases} \tag{5}$$

The flagellar radius a is sufficiently small compared to the length, and the helical shape of the flagellum is denoted by the orthonormal body frame of the center line. The flagellar surface is then modelled as a cylindrical body with radius a from the center line.

In the case of unwrapped mode, the flagellar center line is given by ref[3]:

$$\begin{matrix} \xi_2 = R_H(1 - \exp k_e^2 \xi_1^2) \sin k\xi_1 \\ \xi_3 = R_H(1 - \exp k_e^2 \xi_1^2) \cos k\xi_1 \end{matrix} \quad \text{with } 0 \le \xi_1 \le L_H. \tag{6}$$

ξ_i is the orthonormal body frame with the base of the flagellum as the origin, and R_H and L_H are the helix radius and length, respectively. k is the wave number of the helix, and k_e controls how quickly the helix grows to its maximum amplitude with growing distance from $\xi_1 = 0$.

In the case of wrapped mode, the flagellar center line is given by the following helix equation:

$$\begin{matrix} \xi_2 = R_H \frac{k^2 \xi_1^2}{1 + k^2 \xi_1^2} \sin k\xi_1 \\ \xi_3 = R_H \frac{k^2 \xi_1^2}{1 + k^2 \xi_1^2} \cos k\xi_1 \end{matrix} \quad \text{with } 0 \le \xi_1 \le L_H. \tag{7}$$

The bacterial body and flagellar surface are discretized by 5120 and 4800 triangular meshes, respectively.

C. Numerical procedures

To simulate the bacterial swimming, we consider resistance problems with respect to unknowns $\mathbf{U}$, $\mathbf{\Omega}$, $\boldsymbol{\omega}$, $\mathbf{q}$ and $\mathbf{q}^w$. The cell surface and tube wall are discretized by triangular element and all physical quantities are computed at each vertex. Then, the boundary integral equation (1) is computed by a numerical Gaussian integration scheme[4], and we have the following vector form of Eq. (1):

$$\{\mathbf{v}\} = \{\boldsymbol{\mathcal{T}}_{cc}\}\{\mathbf{q}\} + \{\boldsymbol{\mathcal{T}}_{cw}\}\{\mathbf{q}^w\} \quad \text{when } \mathbf{x} \in \text{cell}, \tag{8}$$

and

$$\{\mathbf{0}\} = \{\boldsymbol{\mathcal{T}}_{wc}\}\{\mathbf{q}\} + \{\boldsymbol{\mathcal{T}}_{ww}\}\{\mathbf{q}^w\} \quad \text{when } \mathbf{x} \in \text{tube surface}. \tag{9}$$

Equation (8) represents the motion of the bacterium and Eq. (9) is the no-slip boundary condition at the tube surface. The vector size of $\{\mathbf{v}\}$ and $\{\mathbf{q}\}$ are $3N$, where N is the total number of nodes on the cell surface. Whereas the size of $\{\mathbf{0}\}$ and $\{\mathbf{q}^w\}$ are $3M$, where M is the number of nodes on the wall. Accordingly, the matrix size of $\{\mathcal{T}_{cc}\}$, $\{\mathcal{T}_{cw}\}$, $\{\mathcal{T}_{wc}\}$ and $\{\mathcal{T}_{ww}\}$ are $3N \times 3N$, $3N \times 3M$, $3M \times 3N$, and $3M \times 3M$, respectively. Substituting Eq. (5) into Eq. (8) and applying the force- and torque-free conditions (Eqs. 3 and 4), the system can be extended to following $(3N + 3M + 9) \times (3N + 3M + 9)$ matrix system:

$$\begin{bmatrix} \mathcal{T}_{cc} & \mathcal{T}_{cw} & \mathcal{V} \\ \mathcal{T}_{wc} & \mathcal{T}_{ww} & \\ & \mathcal{F} & \\ & \mathcal{T} & 0 \\ & \mathcal{T}' & \end{bmatrix} \begin{bmatrix} \mathbf{q} \\ \mathbf{q}^w \\ \mathbf{U} \\ \mathbf{\Omega} \\ \boldsymbol{\omega} \end{bmatrix} = \begin{bmatrix} 0 \\ 0 \\ 0 \\ 0 \\ \mathbf{M}_m \end{bmatrix}. \tag{10}$$

Matrix component $\mathcal{F}$, $\mathcal{T}$, and $\mathcal{T}'$ are computed from Eqs. (3) and (4) with the numerical Gaussian integration, and $\mathcal{V}$ is given by Eq. (5). The dense matrix of (10) is solved with respect to unknowns $\mathbf{q}$, $\mathbf{q}^w$ $\mathbf{U}$, $\mathbf{\Omega}$, and $\boldsymbol{\omega}$ by a lower-upper (LU) factorization technique[4]. Once the translational velocity $\mathbf{U}$ and the angular velocity $\mathbf{\Omega}$ and $\boldsymbol{\omega}$ are given, all material points on the cell surface are updated by a second-order Runge-Kutta method. In addition, the flow field can be calculated by substituting $\mathbf{q}$ and $\mathbf{q}^w$ into Eq. (1).

D. Numerical parameters

Flow simulations are performed in a time-space non-dimensionalised by the fluid viscosity μ, motor torque $|\mathbf{M}_m|$, and body length H. In other words, the length, time, and viscous traction are scaled as $\mathbf{x}^* = \mathbf{x}/H$, $t^* = t|\mathbf{M}_m|/\mu H^3$, and $\mathbf{q}^* = \mathbf{q}H^3/|\mathbf{M}_m|$, respectively. To simulate the bacterial swimming in microchannels, the tube radius R/H is set in the range of 0.2 to 0.34 and the channel length L is set to L/H = 13. If we assume H = 2.5 μm (Fig. S7b), these values are equivalent to R = 0.5 to 0.85 μm and L = 32.5 μm.

The number of elements Ne is set to Ne = 9920 for the cell surface (N = 4964) and Ne = 10008 for the tube wall (M = 5040) so that typical mesh size Δx is smaller than $\Delta x/H = \sqrt{A/Ne} = 0.01$, where A is the surface area. Accuracy of calculating the lubrication flow between the cell body and the wall depends on the mesh size. Thus, a spatial resolution of approximately 25 nm can be calculated with the mesh size of $\Delta x/H = 0.01$.

To mimic the flagellar waveforms observed in experimental measurements, the wave number k is set to kH = 2.5π for the unwrapped mode and kH = 5π for the wrapped mode, respectively, and the helix radius and length are set to R_H/H = 0.1 and L_H/H = 2 for the unwrapped mode and R_H/H = 0.163 and L_H/H = 0.9 for the wrapped mode. The helix growing factor k_e is also set to k_eH = 1, and the flagellar radius a is assumed to be a/H = 0.0125[3].

Time increment Δt for the calculation is set to $\Delta t|\mathbf{M}_m|/\mu H^3 = 0.01$. This corresponds to Δt = 78 μsec by assuming μ = 1 mPa.s, and $|\mathbf{M}_m|$ = 2000 pN.nm.

E. Flow field

The unwrapped mode flow field in free space environment produces an extensional flow in the front to back direction and a suction flow in the lateral direction (Fig. S7c). On the other hand, in the narrow tube, no lateral suction flow is generated and the fluid friction between the cell body and the wall provides strong resistance (Fig. 3g Top). As a result, the flow generated by the flagellum contributes only to the agitation of the fluid around the flagellum and less to its propulsion.

The wrapped mode creates a vortex flow in free space (Fig. S7c), while in the narrow channel the flagellum scrapes the fluid in the gap like a corkscrew, creating a laminar flow structure in the tube and contributing to cell propulsion (Fig. 3g Bottom).

F. Swimming velocity and angular velocity (Fig. 3h and Fig. S7d)

The swimming speed in free space without the tube is $U_0\,\mu H^2/|\mathbf{M}_m| = 0.094$ for the unwrapped mode. This corresponds to $U_0 = 30$ μm/sec, whereas it is 16 μm/sec for the wrapped mode.

In the wrapped mode, the swimming speed is relatively maintained even in narrow channels. In the unwrapped mode, however, the speed decreases monotonically as smaller gap and when $\Delta R/(H/2)$ is less than 0.1, the speed is less than 4% of U_0. We again assume H = 2.5 μm, the gap in a circular tube of 1 micro diameter corresponds to $\Delta R/(H/2) = 0.025$ and the swimming speed is estimated to be U = 180 μm/min for the wrapped mode. This speed is comparable to the experimental measurement (e.g. 104 μm/min using *C. insecticola* in Table S1). In the unwrapped mode, no solution was obtained for conditions below $\Delta R/(H/2) = 0.1$ due to computational instability.

We also calculate the rotational angular velocity in the narrow tube. Effect of small gap on the rotation is relatively small in both modes. Which indicates that the flagellar motor produces sufficient large torque to generate cellular rotation even in the narrow channel.

G. Effect of viscoelasticity

Viscoelastic properties of fluids are an important factor in bacterial movement. The constitutive law in the Maxwell model, which is a linear viscoelastic fluid, is given by using the relaxation time λ of the fluid[5]:

$$\left(1 + \lambda \frac{\partial}{\partial t}\right) \boldsymbol{\tau} = 2\mu \mathbf{D},$$

where $\boldsymbol{\tau}$ is the shear stress of the fluid, μ is the viscosity, and $\mathbf{D}$ is the rate of strain, respectively. Assuming the Stokes flow regime with no inertia, the equation of motion of the Maxwell fluid is described by the boundary integral equation [6]:

$$\mathbf{v}(\mathbf{x}) = -\frac{1}{8\pi\mu}\left(1 + \lambda \frac{\partial}{\partial t}\right) \int \mathbf{J}(\mathbf{x}, \mathbf{y}) \cdot \mathbf{q}(\mathbf{y}) dS(\mathbf{y})$$

where $\boldsymbol{v}$ is the velocity, $\mathbf{J}$ is the Green's function of the Stokeslet, and $\mathbf{q}$ is the traction on the surface.

The viscoelasticity of the Maxwell fluid is controlled by the relaxation time λ, with elasticity dominant for $\lambda >> 1$ and viscosity dominant for $\lambda << 1$ ($\lambda = 0$ behaves as a Newtonian fluid). The relaxation time is nondimensionalized by the Deborah number De, which is defined by using the rotational angular velocity of the flagella Ω as the characteristic time of motion; $De = \lambda\Omega$. Smith et al.[7] estimated the relaxation time for a 1% methylcellulose (M0512, viscosity: 4,000 cP at 2%, Sigma-Aldrich) solution to be 0.006 seconds. This is about 0.04 in terms of Deborah number. In this study, 0.4% methylcellulose (M0512, viscosity: 4,000 cP at 2%, Sigma-Aldrich) was used, and the relaxation time is expected to be shorter. Therefore, the swimming velocity and flow field in the range of $0.001 \leq De \leq 0.05$ are compared with those of Newtonian fluid.

At Deborah numbers around $O(10^{-2})$, the flow structure is similar to that in Newtonian fluid, and the mean swimming velocity is reduced by only about 10%. Since the viscosity of the solution increases dramatically with methylcellulose but the elastic properties are very weak, it can be concluded that there is no qualitative difference between the motion in methylcellulose solutions and that in a Newtonian fluid.

H. Comparison with the previous study

This study adopts modelling that represents short-range interactions, whereas Gidituri *et al*. is based on long-range interactions[8]. The Lorentz reciprocal theorem in the Stokes equation gives solutions in the form of boundary integrals.

$$8\pi\mu\mathbf{v}(\mathbf{x}) = \int \mathbf{J}(\mathbf{x},\mathbf{y}) \cdot \mathbf{q}(\mathbf{y}) dS(\mathbf{y}).$$

This is the exact solution of the Stokes flow, and an infinite series expansion of this solution yields the Stokeslet (zero order moment), Stresslet (first order moment), Rotlet (first order moment) and quadrupole terms (second order moment), with the Stokeslet interacting at $1/r$ acting as the leading-order for the far-field interaction.

$$\int \mathbf{J} \cdot \mathbf{q} dS \sim \mathbf{J} \cdot \mathbf{F} \text{ (Stokeslet)} + \mathbf{K}{:}\, \mathbf{S} \text{ (Stresslet)} + \mathbf{R}{:}\, \mathbf{L} \text{ (Rotlet)} + \mathbf{H}{:}\, \mathbf{Q} \text{ (Quadrupole)} + \cdots.$$

Gidituri *et al*. formulate the flow using Stokeslets[8], where higher order terms above the stresslet are ignored. As a result, there are difficulties with short-range interactions and the accuracy may not be sufficient to resolve the flow in gaps. In this study, the flow is solved in the form of boundary integrals before infinite series expansion, so the effect of the short-range interaction is also included. Therefore, the results may differ where the pipe radius is sufficiently small. In the present study, the swimming velocity in the wrap mode is higher than in the pusher mode, but velocity reversal rarely occurs in Gidituri *et al*.

Supplementary Note 2: Numerical simulations of flagellar dynamics and a hook stiffness

1. Overview of numerical simulation model

A polar flagellated bacterium studied in this work typically has a few flagella at one pole that form a bundle during propulsion. In our mechanical modeling of a flagellar wrapping, we assume the bundle of flagella as a single elastic helical filament. For the deformations of a flexible filament, we compute all the elastic modes of stretching, bending, and twisting, taking full account of the geometric nonlinearities inherent to such slender structures (Fig, S9a), based on the method reported previously[9].

The flagellar filament is connected to a rigid cell body with a flexible hook, a tiny functional mechanical element. The motor torque is transmitted to the filament without loss even when the orientation of the flagellar filament at the base deviates from the rotational axis of the motor. Because the hook is short enough (less than 100 nm) compared to a length of flagellar filament of typically several μm, we take into account effects of the hook flexibility by modelling it as a torsional spring at the connecting point with some stiffness that can be changed independently from that of the flagellar filament. As a crucial hypothesis, we assume short-range attractive interactions between distant segments of a flagellar filament. Such a weakly sticky flagellum assumption is consistent with our observation that a flagellar bundle remains stable in absence of any hydrodynamic effects at stalled rotary motor condition. Finally, the hydrodynamic force from the surrounding fluid is considered at the level of the resistive force theory, meaning that the viscous force is proportional to the local velocity with the local shape-dependent friction coefficients implying the non-local nature of the background viscous flow. In the present composite elastic-rigid model, the bacterial flagellar motor is modelled as a torque dipole embedded at the connecting point.

In the numerical simulation, a helical flagellar filament is discretized into a chain of nodes connected by short segments of approximately constant length a, while the cell body is modelled as a cylindrical rigid body of length $2H$ with spherical end caps of diameter D The total energy of this system, E_{total}, consists of a number of different contributions:

$$E_{\text{total}} = E_{\text{el}} + E_{\text{hook}} + E_{\text{LJ}} + E_{\text{wrap}}, \qquad (1)$$

where E_{el} is the elastic deformation energy of the helical filament that is the sum of the stretching, bending, and twisting energies, E_{hook} is the torsional spring potential that accounts the bending stiffness of the hook segment, E_{LJ} is a truncated Lennard-Jones potential that represents an attractive interaction between the distant nodes as well as the self-avoiding interaction in the filament, and E_{wrap} is the excluded volume interaction potential between the filament and the cell body that defines the wrapping configuration.

The forces and torques acting on each node of the flagellar filament, as well as those on the rigid cell body, can be computed via the variational methods based on the potential energy given in Eq. (1). The bacterial motion is directly driven by the internal motor torque, $\mathbf{M}_m = M_m\mathbf{t}$, where $\mathbf{t}$ defines the axis of the cell body. The flagellar filament is attached at the connecting point $x = \mathbf{R}_B + H\mathbf{t}$, where $\mathbf{R}_B$ represents the center of mass of the cell body. In our discrete model, the motor torque on the filament is decomposed into the orbital and spinning components about the local tangent of the

filament at its base, $\mathbf{u}$. The resulting force $\mathbf{f}_m$ and torque $\mathbf{T}_m$ are applied at the 1st node of the discrete filament. The reaction force and torque on the cell body are given by $\mathbf{f}_m$ and $-(\mathbf{M}_m + H\mathbf{t} \times \mathbf{f}_m)$. We have confirmed both analytically and numerically that all the internal forces and torques, including the motor produced forces and torques, satisfy the action-reaction law, ensuring the overall force and torque free conditions for a freely swimming bacterium. At low Reynolds number relevant to our problem, the positions and orientations of each node of the filament evolve according to the overdamped Langevin equations that describe the translational and rotational motions of the local director frame attached at each node in presence of thermal forcing. Similarly, the viscous force and torque balance equations for the cell body describe the translation of its center of mass position $\mathbf{R}_B$ and the rotation of its body-fixed frame.

We use the Euler iteration method to numerically integrate the appropriately rescaled equations of motion with a nondimensional time step of typically 0.01. The output values are calculated every 10^5–10^6 steps, and the total simulation time is 10^7–10^8 steps. All the nondimensional parameters in the equations are matched with those directly relevant to our experimental observations. Values of some key parameters that may change qualitive behaviors of our numerical model are further explained below. A more detailed description of our mathematical biomechanical modeling will be reported elsewhere.

2. Relevant parameters

A flagellar filament is assumed to have a uniform radius R and pitch P in its natural configuration. In the swimming mode, we set $R_{normal} = 0.2$ μm, $P_{normal} = 2.0$ μm, valid to the Normal form, with the three full turns along the arclength. In the wrapped mode, we set $R_{coil} = 0.6$ μm, $P_{coil} = 1.4$ μm, valid to the Coil form, with the two full turns along the arclength. Note that the both forms are left-handed helices.

We denote the bending, twisting, and stretching moduli of a flagellar filament as A, C and K, respecively. The bending modulus of a bacterial flagellum A has been measured in the previous studies, which on the whole suggest $A = 2.3\text{-}3.5\ \mathrm{pN \cdot \mu m^2}$[10]. In this study, we assume $A = 3.0\ \mathrm{pN \cdot \mu m^2}$ per a filament. In contrast, the twisting modulus C and the stretching modulus K have not been measured directly. Assuming an isotropic elastic rod of diameter a, we choose $Ka^2/A = 16$, which ensures that segment length variations are negligibly small so that the filament is regarded approximately inextensible. For an isotropic elastic rod, we have $C/A = 1/(1+v)$, where v is the Poisson's ratio of the rod material. Since $0 \leq v \leq 1/2$ for ordinary materials, we usually assume $0 \leq C/A \leq 1$. Nevertheless, considering the complexities of the supramolecular structures of a flagellar filament, C/A is allowed to take a wider range of values. We therefore change the twist/bend ratio C/A between 0.5 to 1.0.

On the other hand, we have determined the stiffness of the hook bending torque as $3.36\ \mathrm{pN \cdot \mu m}$ for *C. insecticola* and $16.7\ \mathrm{pN \cdot \mu m}$ for *B. anthina*. Assuming that the bundle analyzed consisted of three identical filaments, we have the stiffness per hook as $k_\theta = 1.12\ \mathrm{pN \cdot \mu m \cdot \mu m}$ for *C.*

insecticola and $k_\theta = 5.54\ \mathrm{pN \cdot \mu m}$ for *B. anthina*. The hook bending modulus, A_{hook}, is related to the stiffness k_θ determined from the main axis angle Brownian motion, as $A_{hook} = k_\theta l$, where l is the hook length. Assuming $l \approx 60\ nm$, we find the rescaled hook stiffness as $A_{hook}/A = 0.02$ for *C. insecticola* and 0.11 for *B. anthina*. In the simulations, we change A_{hook}/A from 0.02 to 0.15.

We set the magnitude of torque generated by the flagellar rotary motor as $M_m =$ $0.40\text{-}0.50 A/R_{coil}$. Assuming $A = 3.0\ \mathrm{pN \cdot \mu m^2}$ and $R_{coil} = 0.6\ \mathrm{\mu m}$, it amounts to $M_m =$ $2000\text{-}2500\ \mathrm{pN \cdot nm}$ per rotary motor, which is only slighly higher than the previously reported values $1300\text{-}1800\ \mathrm{pN \cdot nm}$ for *E. coli*[10].

3. Results

In Fig. S9b-d, we show the three qualitatively distinctive wrapping behaviors observed in the simulations for varying hook/filament stiffness ratio, A_{hook}/A, and the twist/bend stiffness ratio of filament, C/A (Table S2).

For $A_{hook}/A = 0.02$ and $C/A = 0.75$, we find a smooth wrapping transition (Movie S15), in good agreement with the experimental observation (Fig. 4a). For the increased hook stiffness $A_{hook}/A = 0.14$ and $C/A = 0.75$, the wrapping behavior is distinctively different from the soft hook case, where the filament overlaps near the end of the cell body (Movie S16). The tendency is overall unchanged for $C/A = 1.0$. Physically, when the hook is sufficiently stiff, the filament folds before completely wrapping around the cell body, leading to the peculiar morphology dictated probably by the balance between the elasticity and intra-filament attractions. For even smaller twist/bend stiffness ratio given by $C/A = 0.5$ (with the stiff hook of $A_{hook}/A = 0.14$), this feature is more evident, where the filament forms a ring without wrapping around the cell body at all (Movie S17).

These numerical results are all consistent with the optical microscopy observations, which validates our physical modeling of the wrapping bacterium. As a crucial check, the assumption of the sticky flagellar filament will have to be examined in future studies. Overall, our biomechanical modeling and its numerical simulations suggest a potentially important role of the hook stiffness upon the wrapping ability of polar flagellated bacteria.

Supplementary Figures

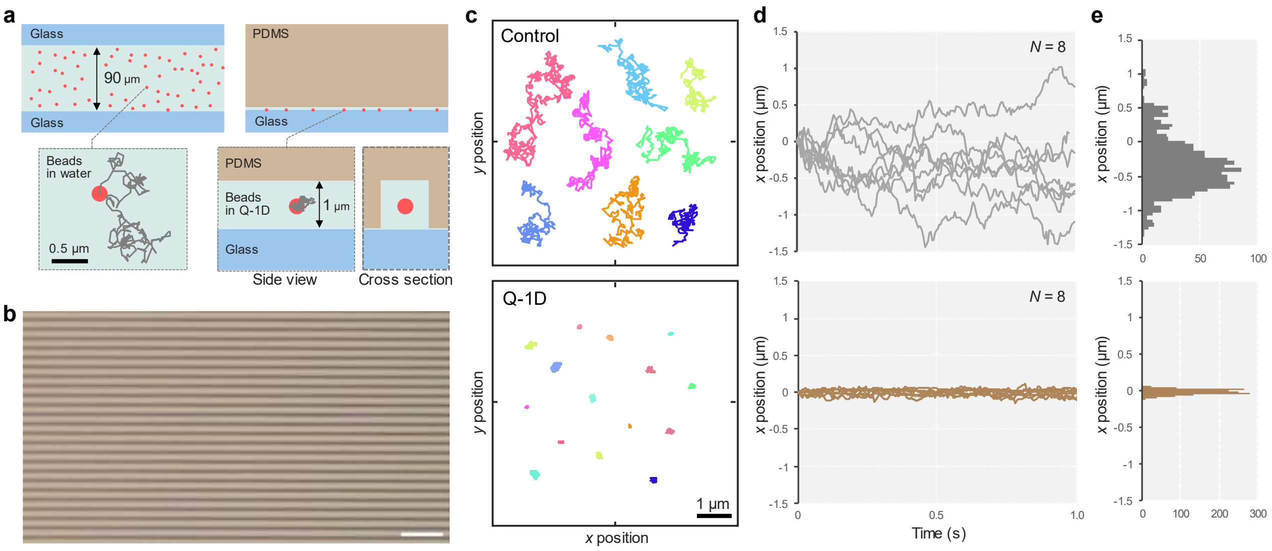

Fig. S1: Brownian motion of microbeads in Q-1D.

a Confinement of microbeads. Schematic of the sample observation from a side view. Fluorescent beads with a diameter of 200 nm suspended in water were used as the sample. Left: Standard chamber. The chamber was assembled with two pieces of coverslip at a height of 90 μm. Right: Q-1D. The sample was confined in the narrow 1×1 μm square tube passage by pressing the device made by PDMS from the top. **b** Phase-contrast image of Q-1D. Line-patterned white area is a space for the sample confinement. Scale Bar, 10 μm. **c** Trajectories of microbeads at 5.6 ms-intervals for 1 s. **d** Time course of the microbeads for 1 sec (*N* = 8 beads). Top: Control as standard chamber. Bottom: Q-1D. **e** Distribution of the bead displacement. Top: standard chamber. Bottom: Q-1D. The variance of the bead displacement in standard chamber and Q-1D are measured as 0.313 and 0.041, respectively. Source data are provided with this paper.

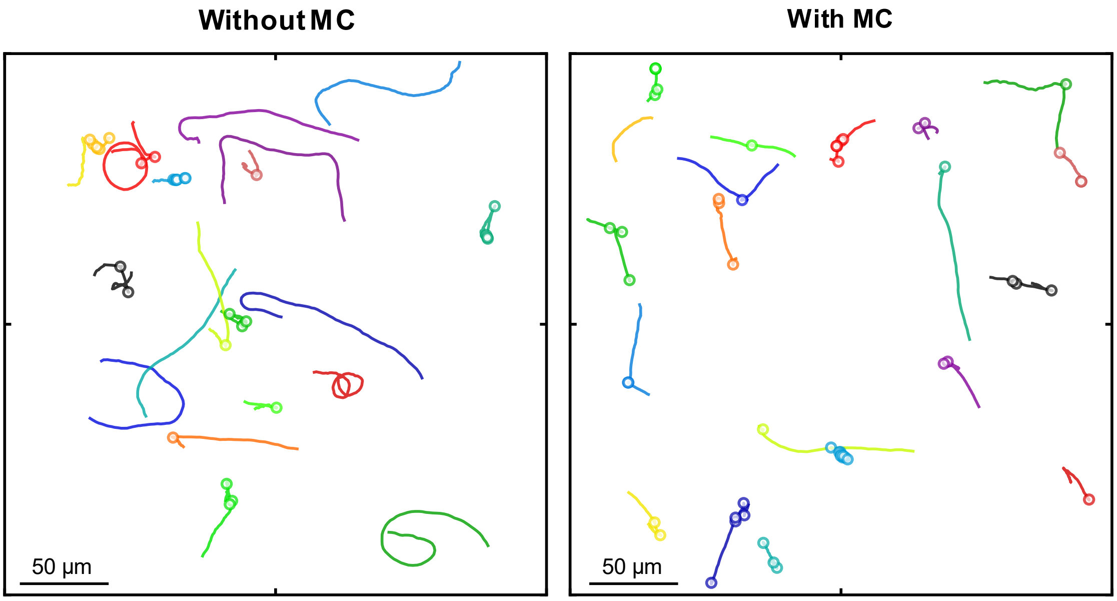

Fig. S2: Swimming pattern of *C. insecticola* in bulk liquid.

Trajectories of the cell at 50 ms-intervals for 4 s in the standard chamber. Left: Without MC. Right: With 0.4% MC. The chamber was assembled with two pieces of coverslip at a height of 90 µm. Directional change of the cells are marked by open circles. Source data are provided with this paper.

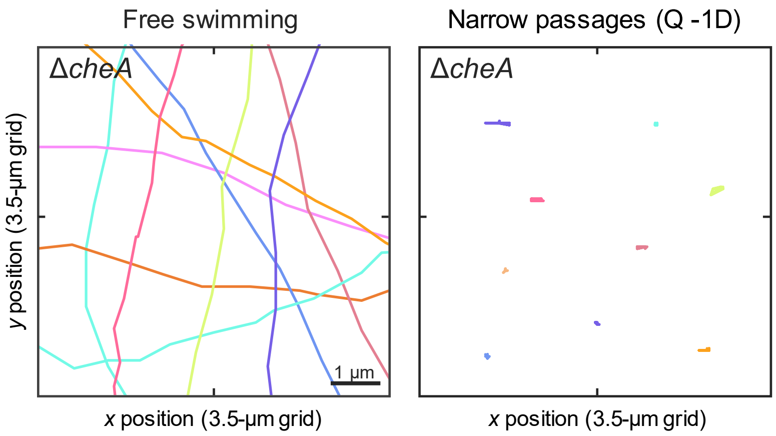

Fig. S3: Cell behavior of Δ*cheA* deletion mutant of *C. insecticola* in Q-1D.
Trajectories of the cell at 50 ms-intervals for 1 s. Left: Free swimming in the standard chamber. The chamber was assembled with two pieces of coverslip at a height of 90 μm. Right: Cell behavior in the narrow passages of Q-1D. Source data are provided with this paper. Source data are provided with this paper.

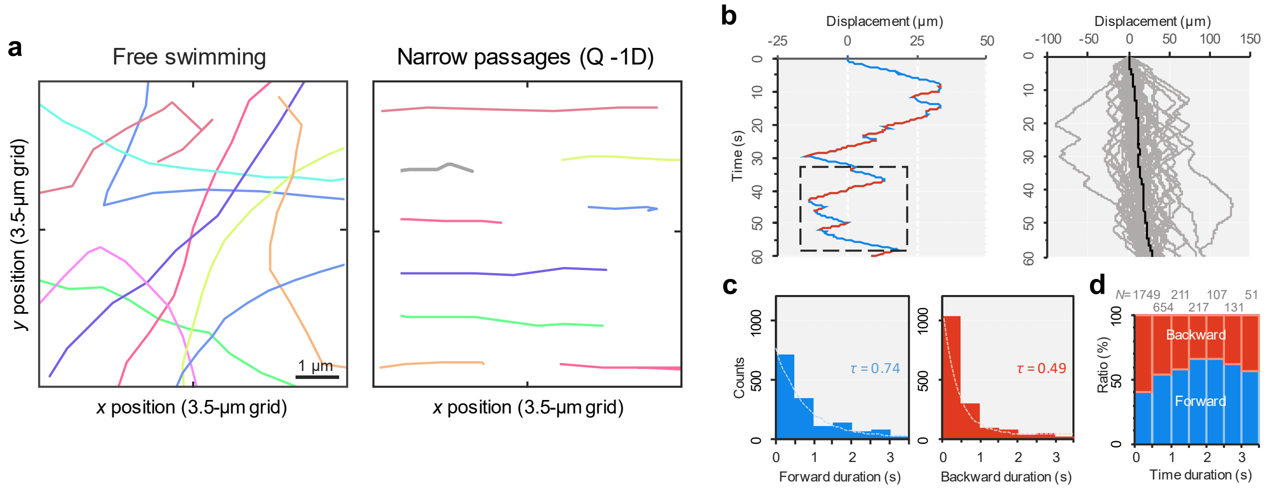

Fig. S4: Cell behavior of *S. enterica* in Q-1D.

a Trajectories of the cell for 1 s in the standard chamber (left) and narrow passages of Q-1D (right). **b** Time course of cell displacement in Q-1D. Left: Single cell. Forward and backward movements are represented as blue and red colored lines respectively. Right: Overlays of 50 cells and the average. **c** Distribution of the forward and backward time duration. The duration from a directional change to the next were measured in Q-1D. Color code the same way as (**b**). Dashed lines show the fit of single exponential decay, where time constant τ is presented. **d** Ratio of the time duration of forward and backward direction. The duration of each run was measured, and the number of forward versus backward runs was counted. The ratio of forward runs was then calculated for runs with durations falling into 0.5 s intervals. The sample number of each bin are presented at the top. Source data are provided with this paper.

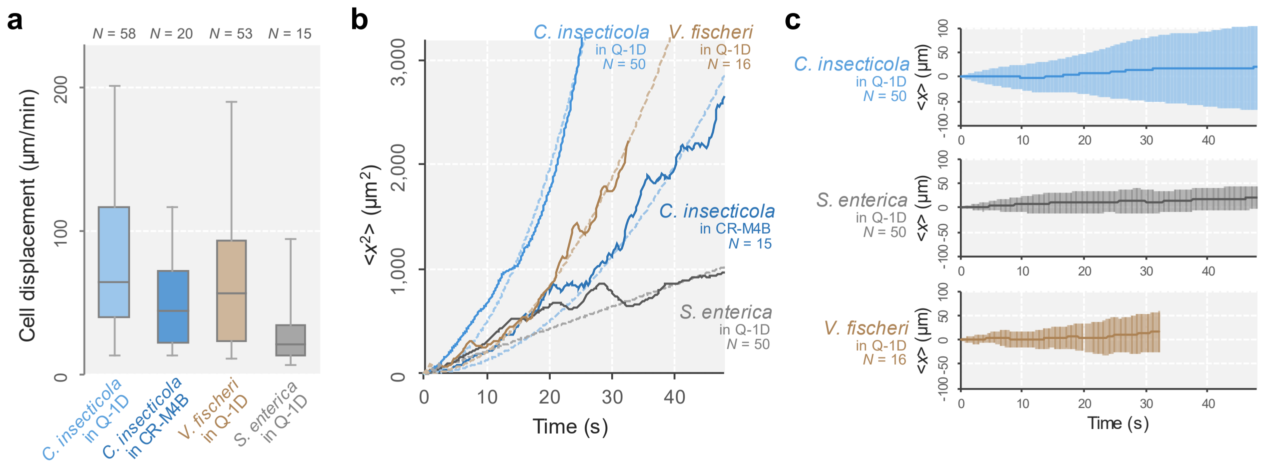

Fig. S5: Cell displacement in a narrow passage.

a Net displacement of *C. insecticola* in Q-1D, *C. insecticola* in CR-M4B region in host (the same data set from Fig. 1d), *V. fischeri* in Q-1D, and *S. enterica* in Q-1D for 1 min. Box plot presents the minimum, maximum, sample median, and the first and third quartiles. **b** MSD plots of cell movement. Dashed lines of *C. insecticola* and *V. fischeri* represents a hyperbolic fitting, $<x^2> = at^2$. Dashed lines of *S. enterica* represents a linear fitting, $<x^2> = at$. **c** The signed mean displacement plots of cell movement. Time course of cell displacement in Q-1D, where rightward motion is defined as positive and leftward as negative along the *x* axis. Source data are provided with this paper.

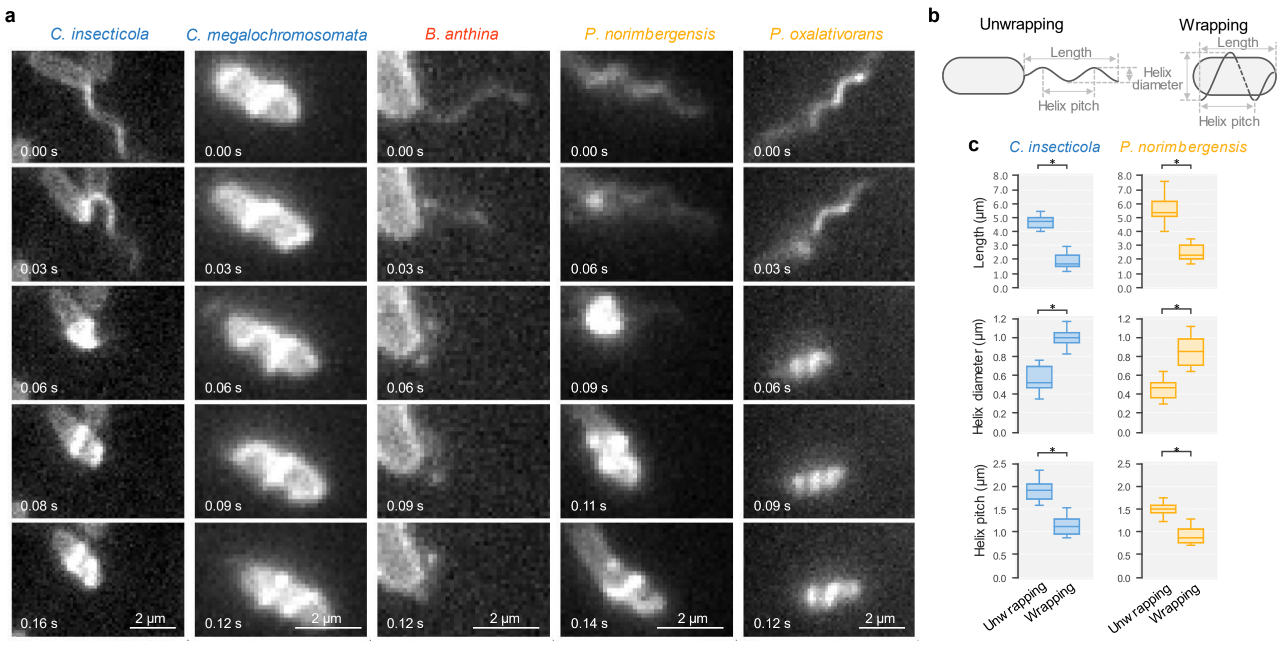

Fig. S6: Visualization of flagellar filament in swimming cells of *Burkholderia sensu lato* group. **a** Sequential fluorescent images of *C. insecticola*, *C. megalochromosomata*, *B. anthina*, *P. norimbergensis*, and *P. oxalativorans*. The swimming mode was changing from unwrapping to wrapping in *C. insecticola*, *P. norimbergensis*, and *P. oxalativorans*. The *C. megalochromosomata* cell propels itself with flagellar wrapping. The *B. anthina* cell is captured at incomplete flagellar wrapping when the flagellar filaments are folded as a ring at the proximal end. All observations are performed in the presence of 0.5% MC. **b** Schematic of the parameter of the helix morphology in unwrapping (right) and wrapping cells (left). **c** Measurements of the helix morphology of flagellar filaments in *C. insecticola*, *P. norimbergensis*. Box plots of flagellar length along the helix axis, a helix radius, and a helix pitch. These parameters were measured based on the fluorescent image of their flagellar filament in Q-1D (N = 20 cells). Statistical analysis was performed using the unpaired t test ($p < 0.05$). Source data are provided with this paper.

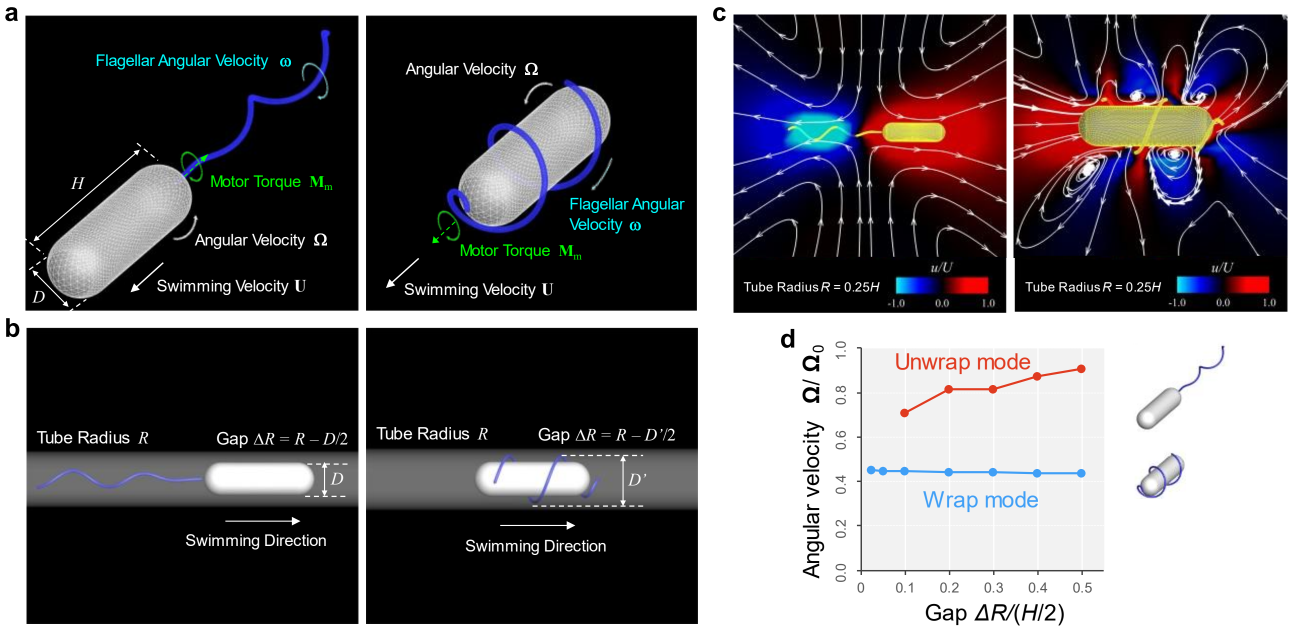

Fig. S7: Numerical simulations of a bacterium swimming in a narrow tube.
a Fluid mechanical model of swimming bacteria exhibiting two distinct modes. Left: Unwrapped mode. Right: Wrapped mode. The cell body is modelled as a cylinder of length H with spherical ends of diameter D. The flagellum has a helical shape with the helical diameter and pitch set to match experimental measurements (Fig. S6bc). The flagellum is assumed to be driven by the motor torque $\mathbf{M}_m$ at the root of the flagellum, and the rigid body translation velocity $\mathbf{U}$ and the angular velocities $\mathbf{\Omega}$, and $\boldsymbol{\omega}$ are derived from the boundary integral equation of the Stokes flow. The cell body and the flagellar surface are discretized with 5120 and 4800 triangular meshes, respectively. **b** Problem setting of the numerical simulation. Left: Unwrapped mode. Right: Wrapped mode. The bacterial model in a circular tube of radius R swims in one direction and the minimum distance between the wall and the bacterium is defined as ΔR. **c** Time-averaged flow field around the bacterial model in free space. Left: Unwrapped mode. Right: Wrapped mode. White arrows are the streamline and contour color indicate the velocity component in the swimming direction normalized by the swimming velocity $\mathbf{U}$. **d** Angular velocity $\mathbf{\Omega}$ as a function of ΔR. $\mathbf{\Omega}_0$ is the angular velocity of the unwrap mode in a free space. Source data are provided with this paper.

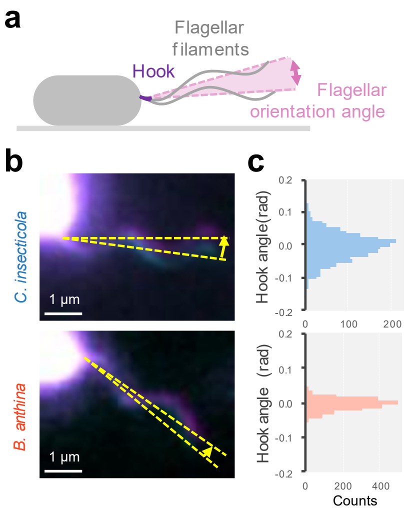

Fig. S8: Fluctuation of flagellar filaments at the proximal part.

a Schematic. Flagellar rotation was inactivated by CCCP, and the cell was immobilized on a glass surface. **b** Fluorescent images. Two sequential images are colored by magenta and cyan, respectively, and merged to see the fluctuation. Dashed yellow line is the helix axis of the flagellar filaments. **c** Distribution of the flagellar orientation angle for a single cell recorded at 5 ms for 10 s. Hook flexibility is measured by the variance of the flagellar orientation angle (see Methods). Homogeneity of variances was analyzed by Bartlett test ($p < 0.05$). Source data are provided with this paper.

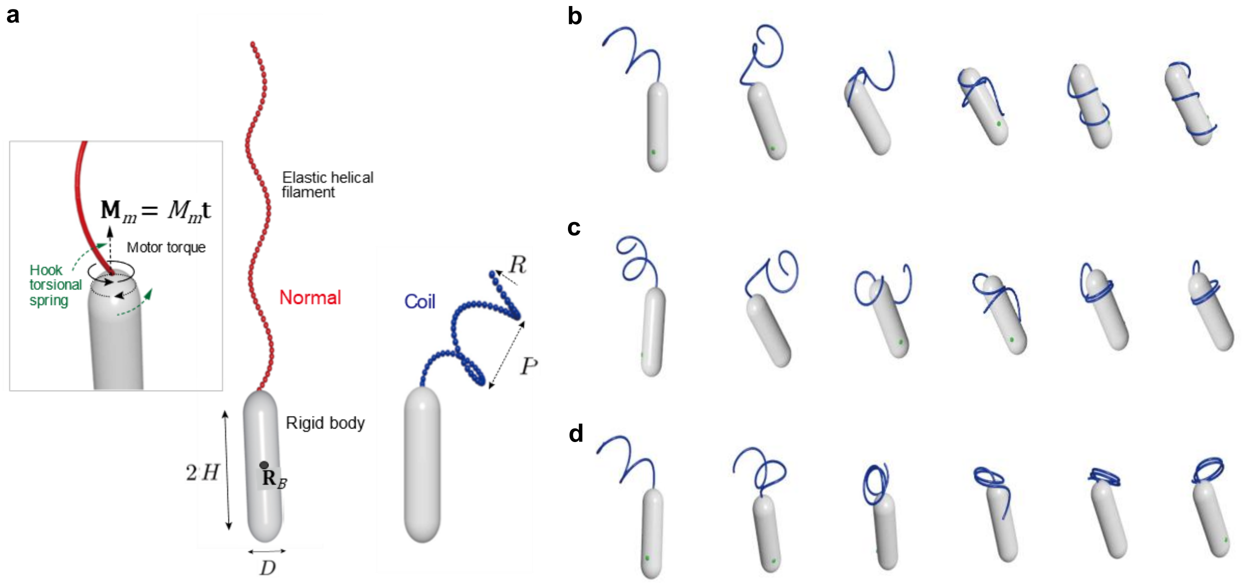

Fig. S9: Numerical simulations of flagellar dynamics and a hook stiffness.

a Numerical simulation model. Schematics of the model for wrapping bacterium and some relevant geometric parameters. A set of discrete points representing a flagellar filament is explicitly drawn here. The number of the nodes is set $N = 60$. Inset shows a close-up view of the connecting point, where the torques by the flagellar motor and by the hook stiffness apply to the filament base. Note that reaction torques also apply to the cell body. **b-d** Typical snapshots from our numerical simulations. For all three cases, the attractive interaction between distant segments in the filament is assumed, with the Lennard-Jones potential parameters $\epsilon_{\mathrm{LJ}} = 20k_BT$ and $r_{\mathrm{cutoff}} = 2a$, where k_BT is the thermal energy. **b** Normal wrapping for $C/A = 0.75$ and $A_{hook}/A = 0.02$ (soft hook). **c** Incomplete wrapping for $C/A = 0.75$ and $A_{hook}/A = 0.14$ (stiff hook). **d** Ring formation for $C/A = 0.50$ and $A_{hook}/A = 0.14$ (stiff hook). Note that the cell body also translates during the wrapping, but its positions are shifted to align for the visualization purpose here.

Supplementary Tables

Table S1: Cell motility of 13 species in *Burkholderia* sensu lato and allied groups

Species	Flagellation*	Swimming speed in standard chamber (μm/s) †	Cell dis-placement in Q-1D (μm/min) ‡	Infection rate (%) §	Flagellar wrapping ¶
Caballeronia insecticola	lophotrichous[11-13]	26.4 ± 8.4	104 ± 52	100	Yes
Caballeronia megalochromosomata	monotrichous[12-14]	22.4 ± 5.8	132 ± 92	100	Yes
Caballeronia glathei	monotrichous or lophotrichous[12,13,15]	22.1 ± 9.5	86 ± 47	100	n.d.
Caballeronia telluris	monotrichous or lophotrichous[12,13,16]	19.6 ± 6.9	42 ± 29	100	n.d.
Paraburkholderia tropica	monotrichous or lophotrichous[17]	34.1 ± 12.4	114 ± 83	100	n.d.
Paraburkholderia mimosarum	monotrichous or lophotrichous[13,18]	28.0 ± 6.0	0 ± 0	0	n.d.
Paraburkholderia tuberum	monotrichous or lophotrichous[13,19]	23.0 ± 5.3	0 ± 0	0	n.d.
Paraburkholderia graminis	monotrichous or lophotrichous[15]	34.1 ± 10.5	78 ± 58	100	n.d.
Burkholderia plantarii	monotrichous or lophotrichous[20]	23.4 ± 7.9	6 ± 6	0	n.d.
Burkholderia anthina	monotrichous or lophotrichous[13,21]	14.7 ± 4.8	0 ± 0	10	No
Pandoraea oxalativorans	monotrichous[22,23]	20.9 ± 7.6	43 ± 27	100	Yes
Pandoraea norimbergensis	monotrichous[23]	19.8 ± 3.8	61 ± 35	100	Yes
Cupriavidus taiwanensis	peritrichous[24]	21.7 ± 9.3	0 ± 0	0	n.d.
Salmonella enterica	peritrichous	22.8 ± 5.2	25 ± 10	n.d.	No

*The number and arrangement of flagella on a bacterial cell.

†Swimming speed was measured in the growth medium of each bacterium.

‡Cell displacement was measured in the growth medium with 0.4% MC.

§Infection rate was measured by the number of infected stink bugs/total number of investigated stink bugs[25].

¶Flagellar wrapping was examined with the species that can be labeled the flagellar filaments by amine-reactive fluorescent dye. The species that cannot visualized the flagellar filaments were presented as not detected (n.d.).

Table S2. Details of the simulation data

Mode	$A_{\mathrm{hook}}/A^{\dagger}$	$C/A^{\ddagger}$	Motor	Flagellar form	Movie
Wrapping	0.02 (soft)	0.75	0.40 CW	Coil	Movie S15
Incomplete wrapping	0.14 (stiff)	0.75	0.40 CW	Coil	Movie S16
Ring formation	0.14 (stiff)	0.50	0.40 CW	Coil	Movie S17

† A_{hook}/A: the ratio of the hook bending stiffness to that of the flagellum.

‡ C/A: twist to bend ratio of flagellum.

Motor torque $\mathbf{M}_m$ is shown in a unit of A/R_{coil}.

All the other simulation parameters will be provided in Supplemental Note 2.

Attraction parameter (LJ potential): $\epsilon_{\mathrm{LJ}} = 20k_BT,\ r_{\mathrm{cutoff}} = 2a$.

Table S3. Culture condition of bacterial species in this study

Species	Medium	Temperature (°C)
Caballeronia insecticola (RPE64) JCM 31142	YG	28
Caballeronia megalochromosomata DSM 100850	YG	28
Caballeronia glathei JCM 10563	YG	28
Caballeronia telluris LMG 22936	YG	28
Paraburkholderia tropica DSM 15359	YG	28
Paraburkholderia mimosarum DSM 21841	YG	28
Paraburkholderia tuberum DSM 18489	YG	28
Paraburkholderia graminis DSM 17151	YG	28
Burkholderia plantarii JCM 5492	YG	28
Burkholderia anthina DSM 16086	YG	28
Pandoraea oxalativorans DSM 23570	YG	28
Pandoraea norimbergensis JCM 10565	YG	28
Cupriavidus taiwanensis DSM17343	YG	28
Salmonella enterica serovar Typhimurium strain SJW1103	LB	37
Vibrio fischeri (*Aliivibrio fischeri*) ATCC7744	SWT	28

Table S4. Oligonucleotides used in this study

Purpose	Primer Name	Target	Sequence (5'-3')[a,b]	Cloning strategy
C. inseticola genome *flgE* knockout	Cins_flgE_LF	Lower reigion of *flgE* of *C. insecticola*	GAT**GAATTC**GCGTCTGCAACATCTGGTCG	Restriction cloning into the EcoRI and HindIII sites of pK18mobsacB
	Cins_flgE_LR		ATA**GGATCC**CAATCTGTAAGCACCGCCCG	
	Cins_flgE_RF	Upper reigion of *flgE* of *C. insecticola*	CATA**GGATCC**GCGCTCGCCCTATAAAA	
	Cins_flgE_RR		GAT**AAGCTT**GAACGTCACCGCGTCGAATC	
B. anthina genome *flgE* knockout	Bant_flgE_LF	Lower reigion of *flgE* of *B. anthina*	GCGGCCTCGTCGACCAGACGCTCATCAA	Seamless cloning into the BamHI site of pK18mobsacB
	Bant_flgE_LR		CAGGTCGACTCTAGAGATCAGCACCTGGAAGAAGCC	
	Bant_flgE_RF	Upper reigion of *flgE* of *B. anthina*	AGCTCGGTACCCGGGCTGCACTACGAAACCGGGAT	
	Bant_flgE_RR		TGGTCGACGAGGCCGCTCAGACCCT	
Plasmid for *flgE*$_{Ci}$ expression	Cins_flgE_comp_F	*flgE* and upstream reigion of *C. insecticola*	CCTTGCGTATAATATTTGCCTCGATTCGAAGGGCACGG	Seamless cloning into the NcoI site of pBBR122 vector
	Cins_flgE_comp_R		TTCTTCGCCCCCGTTTTCACTTACAGATTGATCAGCGTCTGG	
Plasmid for *flgE*$_{Ba}$ expression	Bant_flgE_comp_F	*flgE* and upstream reigion of *B. anthina*	CCTTGCGTATAATATTTGCCGACAGCACCGGCACGCAG	
	Bant_flgE_comp_R		TTCTTCGCCCCCGTTTTCACTTACAGGTTGATGAGCGTCTGGTC	

[a] Restriction sites are indicated in bold.

[b] Homology arms for seamless cloning are indicated by underlines.

Table S5. Strains and mutants used in this study

Strains	Description	Reference
Caballeronia insecticola RPE64	Wild type	Ref[26]
C. insecticola Δ*cheA*	Chemotaxis deletion mutant. No switchback.	Ref[27]
C. insecticola GFP	GFP expressing cell	Ref[28]
C. insecticola Δ*flgE*	Gene coding hook protein deletion mutant	This study
C. insecticola Δ*flgE* + $flgE_{Ci}$	Complement with *C.insecticola flgE*	This study
C. insecticola Δ*flgE* + $flgE_{Ba}$	Complement with *B. anthina flgE*	This study
Burkholderia anthina DSM 16086	Wild type	Ref[25]
B. anthina Δ*flgE*	Gene coding hook protein deletion mutant	This study
B. anthina Δ*flgE* + $flgE_{Ba}$	Complement with *B. anthina flgE*	This study
B. anthina Δ*flgE* + $flgE_{Ci}$	Complement with *C. insecticola flgE*	This study

Supplementary References

1 Purcell, E. M. Life at low Reynolds number. *Am J Phys* **45**, 3-11 (1977). https://doi.org/10.1119/1.10903

2 Pozrikidis, C. *Boundary integral and singularity methods for linearized viscous flow*. (Cambridge Univ Press, 1992).

3 Kanehl, P. & Ishikawa, T. Fluid mechanics of swimming bacteria with multiple flagella. *Phys Rev E* **89**, 042704 (2014). https://doi.org/10.1103/PhysRevE.89.042704

4 Omori, T. & Ishikawa, T. Upward swimming of a sperm cell in shear flow. *Phys Rev E* **93**, 032402 (2016). https://doi.org/10.1103/PhysRevE.93.032402

5 Lubkin, D. J., Gaffney, E. A. & Blake, J. R. A viscoelastic traction layer model of muco-ciliary transport. *Bull Math Biol* **69**, 289-327 (2007). https://doi.org/10.1007/s11538-005-9036-x

6 Omori, T. & Ishikawa, T. Swimming of Spermatozoa in a Maxwell Fluid. *Micromachines* **10**, 78 (2019).

7 Smith, D. J., Gaffney, E. A., Gadêlha, H., Kapur, N. & Kirkman-Brown, J. C. Bend propagation in the flagella of migrating human sperm, and its modulation by viscosity. *Cell Motil Cytoskeleton* **66**, 220-236 (2009). https://doi.org/10.1002/cm.20345

8 Gidituri, H., Ellero, M. & Balboa Usabiaga, F. Swimming efficiently by wrapping. *J Fluid Mech* **993**, A7 (2024). https://doi.org/10.1017/jfm.2024.594

9 Wada, H. & Netz, R. R. Discrete elastic model for stretching-induced flagellar polymorphs. *Euro Phys Lett* **82**, 28001 (2008). https://doi.org/10.1209/0295-5075/82/28001

10 Nord, A. L. *et al.* Dynamic stiffening of the flagellar hook. *Nat Commun* **13**, 2925 (2022). https://doi.org/10.1038/s41467-022-30295-7

11 Takeshita, K. *et al. Burkholderia insecticola* sp. nov., a gut symbiotic bacterium of the bean bug *Riptortus pedestris*. *Int J Syst Evol Microbiol* **68**, 2370-2374 (2018). https://doi.org/https://doi.org/10.1099/ijsem.0.002848

12 Dobritsa, A. P. & Samadpour, M. Transfer of eleven species of the genus *Burkholderia* to the genus *Paraburkholderia* and proposal of *Caballeronia* gen. nov. to accommodate twelve species of the genera *Burkholderia* and Paraburkholderia. *Int J Syst Evol Microbiol* **66**, 2836-2846 (2016). https://doi.org/https://doi.org/10.1099/ijsem.0.001065

13 Sawana, A., Adeolu, M. & Gupta, R. S. Molecular signatures and phylogenomic analysis of the genus *Burkholderia*: proposal for division of this genus into the emended genus *Burkholderia* containing pathogenic organisms and a new genus *Paraburkholderia* gen. nov. harboring environmental species. *Front Genet* **Volume 5 - 2014** (2014). https://doi.org/10.3389/fgene.2014.00429

14 Baek, I. *et al. Burkholderia megalochromosomata* sp. nov., isolated from grassland soil. *Int J Syst Evol Microbiol* **65**, 959-964 (2015). https://doi.org/https://doi.org/10.1099/ijs.0.000046

15 Viallard, V. *et al. Burkholderia graminis* sp. nov., a rhizospheric *Burkholderia* species, and reassessment of [*Pseudomonas*] *phenazinium*, [*Pseudomonas*] *pyrrocinia* and [*Pseudomonas*] *glathei*

as *Burkholderia*. *Int J Syst Evol Microbiol* **48**, 549-563 (1998). https://doi.org/https://doi.org/10.1099/00207713-48-2-549

16 Vandamme, P. *et al. Burkholderia humi* sp. nov., *Burkholderia choica* sp. nov., *Burkholderia telluris* sp. nov., *Burkholderia terrestris* sp. nov. and *Burkholderia udeis* sp. nov.: *Burkholderia glathei*-like bacteria from soil and rhizosphere soil. *Int J Syst Evol Microbiol* **63**, 4707-4718 (2013). https://doi.org/https://doi.org/10.1099/ijs.0.048900-0

17 Reis, V. M. *et al. Burkholderia tropica* sp. nov., a novel nitrogen-fixing, plant-associated bacterium. *Int J Syst Evol Microbiol* **54**, 2155-2162 (2004). https://doi.org/https://doi.org/10.1099/ijs.0.02879-0

18 Chen, W.-M. *et al. Burkholderia mimosarum* sp. nov., isolated from root nodules of *Mimosa* spp. from Taiwan and South America. *Int J Syst Evol Microbiol* **56**, 1847-1851 (2006). https://doi.org/https://doi.org/10.1099/ijs.0.64325-0

19 Vandamme, P., Goris, J., Chen, W.-M., de Vos, P. & Willems, A. *Burkholderia tuberum* sp. nov. and *Burkholderia phymatum* sp. nov., nodulate the roots of tropical legumes. *Syst Appl Microbiol* **25**, 507-512 (2002). https://doi.org/https://doi.org/10.1078/07232020260517634

20 Azegami, K. *et al. Pseudomonas plantarii* sp. nov., the causal agent of rice seedling blight. *Int J Syst Evol Microbiol* **37**, 144-152 (1987). https://doi.org/https://doi.org/10.1099/00207713-37-2-144

21 Vandamme, P. *et al. Burkholderia anthina* sp. nov. and *Burkholderia pyrrocinia*, two additional *Burkholderia cepacia* complex bacteria, may confound results of new molecular diagnostic tools. *FEMS Immunol Med Microbiol* **33**, 143-149 (2002). https://doi.org/10.1111/j.1574-695X.2002.tb00584.x

22 Sahin, N. *et al. Pandoraea oxalativorans* sp. nov., *Pandoraea faecigallinarum* sp. nov. and *Pandoraea vervacti* sp. nov., isolated from oxalate-enriched culture. *Int J Syst Evol Microbiol* **61**, 2247-2253 (2011). https://doi.org/https://doi.org/10.1099/ijs.0.026138-0

23 Coenye, T. *et al.* Description of *Pandoraea* gen. nov. with *Pandoraea apista* sp. nov., *Pandoraea pulmonicola* sp. nov., *Pandoraea pnomenusa* sp. nov., *Pandoraea sputorum* sp. nov. and *Pandoraea norimbergensis* comb. nov. *Int J Syst Evol Microbiol* **50**, 887-899 (2000). https://doi.org/https://doi.org/10.1099/00207713-50-2-887

24 Vaneechoutte, M., Kämpfer, P., De Baere, T., Falsen, E. & Verschraegen, G. *Wautersia* gen. nov., a novel genus accommodating the phylogenetic lineage including *Ralstonia eutropha* and related species, and proposal of *Ralstonia* [*Pseudomonas*] *syzygii* (Roberts *et al*. 1990) comb. nov. *Int J Syst Evol Microbiol* **54**, 317-327 (2004). https://doi.org/https://doi.org/10.1099/ijs.0.02754-0

25 Itoh, H. *et al.* Host–symbiont specificity determined by microbe–microbe competition in an insect gut. *Proc Natl Acad Sci U S A* **116**, 22673-22682 (2019). https://doi.org/doi:10.1073/pnas.1912397116

26 Kikuchi, Y., Hosokawa, T. & Fukatsu, T. Insect-microbe mutualism without vertical transmission: a stinkbug acquires a beneficial gut symbiont from the environment every generation. *Applied and environmental microbiology* **73**, 4308-4316 (2007). https://doi.org/10.1128/aem.00067-07

27 Ohbayashi, T. *et al.* Insect's intestinal organ for symbiont sorting. *Proc Natl Acad Sci U S A* **112**, E5179-E5188 (2015). https://doi.org/doi:10.1073/pnas.1511454112

28 Kikuchi, Y. & Fukatsu, T. Live imaging of symbiosis: spatiotemporal infection dynamics of a GFP-labelled *Burkholderia* symbiont in the bean bug *Riptortus pedestris*. *Mol Ecol* **23**, 1445-1456 (2014). https://doi.org/10.1111/mec.12479
