# Supplementary material for: Bacteria break through one-micrometer-square passages by flagellar wrapping
Source: Nat Commun. 2026 Jan 20;17:713. doi: 10.1038/s41467-025-67507-9 (PMC12820085; doi:10.1038/s41467-025-67507-9)
Supplement: Supplementary file 2 — Description of Additional Supplementary Files [file 41467_2025_67507_MOESM2_ESM.pdf]

Description of Additional Supplementary Files:

Supplementary Movie 1: Behavior of *C. insecticola* in the CR of *R. pedestris*. The second instar nymphs were fed with a suspension of GFP expressing *C. insecticola*. The symbiotic organs of the nymphs were dissected 2 hours after feeding. M4 region is located at the right of the movie.

Supplementary Movie 2: Flagellar dynamics of WT C. insecticola cells in the midgut of *R. pedestris*. The second instar nymphs were fed a suspension of symbiotic bacteria cells with fluorescently labeled body and flagellar filaments. The symbiotic organs of the nymphs were dissected 2 hours after feeding. M4 region is located at the upper right of the movie. Yellow arrows indicate flagellar wrapping cells. Area 26.0 μm × 19.5 μm.

Supplementary Movie 3: Cell behavior of wild type *C. insecticola* in Q-1D captured with phase-contrast microscopy. Narrow passages of the Q-1D were align parallel to the horizontal axis. Area 300 μm × 300 μm.

Supplementary Movie 4: Cell behavior of Δ*cheA* mutant of *C. insecticola* in Q-1D captured with phase-contrast microscopy. Area 300 μm × 300 μm.

Supplementary Movie 5: Cell behavior of *S. enterica* in Q-1D captured with phase-contrast microscopy. Area 300 μm × 300 μm.

Supplementary Movie 6: Cell behavior of *V. fischeri* in Q-1D captured with phase-contrast microscopy. Area 300 μm × 300 μm.

Supplementary Movie 7: Cell behavior of *P. norimbergensis* in Q-1D captured with phase-contrast microscopy. Area 300 μm × 300 μm.

Supplementary Movie 8: Cell behavior of *B. anthina* in Q-1D captured with phase-contrast microscopy. Area 300 μm × 300 μm.

Supplementary Movie 9: Cell behavior and flagellar dynamics of 5 species of Burkholderia sensu lato group. *C. insecticola*, *C. megalochromosomata*, *B. anthina*, *P. norimbergensis*, and *P. oxalativorans* cells were labeled by fluorescent dye, suspended in the liquid medium containing 0.5% MC and captured with fluorescent microscopy at 5-ms interval. Area 35.1 μm × 31.2 μm.

Supplementary Movie 10: Visualization of flagellar filaments of *C. insecticola* cell in Q-1D. Cells were labeled by fluorescent dye, suspended in the liquid medium containing 0.4% MC and captured with fluorescent microscopy at 12.5-ms interval. Area 32.9 μm × 5.2 μm.

Supplementary Movie 11: Dynamics of flagellar filaments of *S. enterica* in Q-1D. Cells were labeled by fluorescent dye, suspended in the buffer containing 0.4% MC and captured with fluorescent microscopy at 10-ms interval. The movie consists of two parts: the first half shows only the fluorescence signal, highlighting the flagellar dynamics during cell swimming along the channel; the second half presents the same sequence with the magenta overlay, indicating the channel boundaries. Area 33.3 μm × 8.8 μm.

Supplementary Movie 12: Dynamics of flagellar filaments of *C. insecticola* in Q-1D. Cells were labeled by fluorescent dye, suspended in the buffer containing 0.4% MC and captured with fluorescent microscopy at 20-ms interval. The movie consists of two parts: the first half shows only the fluorescence signal, highlighting the flagellar dynamics during cell swimming along the channel; the second half presents the same sequence with the magenta overlay, indicating the channel boundaries. Area 18.6 μm × 3.9 μm.

Supplementary Movie 13: Comparison of the dynamics of flagellar filaments in Q-1D. The cells of *C. insecticola* WT and Δ*cheA* mutant cells were labeled by fluorescent dye, suspended in the liquid medium containing 0.4% MC and captured with fluorescent microscopy. Area of each real movie 32.9 μm × 5.2 μm.

Supplementary Movie 14: Fluctuation of flagellar filaments. Flagellar rotation was inactivated by CCCP, and the cell was immobilized on a glass surface. Flagellar filaments were wobbled at the proximal end with small angle due to Brownian motion. Cells were labeled with aminereactive fluorescent dye to see the flagellar filaments, and captured at 5-ms interval. Area 10.4 μm × 3.9 μm.

Supplementary Movie 15: Numerical simulations of flagellar wrapping. The attractive interaction between distant segments in the filament is assumed, with the LennardJones potential parameters $\epsilon_{\mathrm{LJ}} = 20k_BT$ and $r_{\mathrm{cutoff}} = 2a$, where k_BT is the thermal energy. The parameters of C/A = 0.75 and A_{hook}/A = 0.02 are used as a soft hook.

Supplementary Movie 16: Numerical calculation of incomplete flagellar wrapping. The attractive interaction between distant segments in the filament is assumed, with the Lennard-Jones potential parameters $\epsilon_{\mathrm{LJ}} = 20k_BT$ and $r_{\mathrm{cutoff}} = 2a$, where k_BT is the thermal energy. The parameters of C/A = 0.75 and A_{hook}/A = 0.14 are used as a stiff hook.

Supplementary Movie 17: Numerical calculation of flagellar ring formation. The attractive interaction between distant segments in the filament is assumed, with the LennardJones potential parameters ϵ_{LJ} = $20k_BT$ and r_{cutoff} = $2a$, where k_BT is the thermal energy. The parameters of C/A = 0.50 and A_{hook}/A = 0.14 are used as a stiff hook.

Supplementary Movie 18: Comparison of numerical calculation and real images. Numerical results for rigid and flexible hook (see also Movie S15 and Movie S17) were presented with the dynamics of flagellar filaments in *C. insecticola* and *B. anthina*. The cells were labeled by fluorescent dye, suspended in the liquid medium containing 0.5% MC, and flagellar filaments were captured with fluorescent microscopy at 5-ms interval for 0.5 s (see also Movie S9). Area of each real movie 7.80 μm × 5.85 μm.

Supplementary Movie 19: Visualization of flagellar filaments of *B. anthina* with $FlgE_{Ci}$. Cells were labeled by fluorescent dye, suspended in the liquid medium containing 0.4% MC and captured with fluorescent microscopy at 5-ms interval. Area 10.4 μm × 7.8 μm.
